# Community violence and internalizing mental health symptoms in adolescents: A systematic review.

**DOI:** 10.1186/s12888-022-03873-8

**Published:** 2022-04-09

**Authors:** Claudia Reis Miliauskas, Daniela Porto Faus, Valéria Lima da Cruz, João Gabriel Rega do Nascimento Vallaperde, Washington Junger, Claudia Souza Lopes

**Affiliations:** 1 Medical Sciences College/Department of Medical Specialties, State University of Rio de Janeiro, Vinte e Oito de Setembro Avenue, 77, 4° floor, 432. Vila Isabel, Rio de Janeiro, 20.551-030 Brazil; 2 Institute of Social Medicine/State University of Rio de Janeiro, São Francisco Xavier Street, 524, Maracanã, 7 floor, Rio de Janeiro, 20.550-013 Brazil; 3 Universidade Federal do Rio de Janeiro, Maternidade Escola, Laranjeiras Street, 180. Laranjeiras, Rio de Janeiro, 22.240-000 Brazil

**Keywords:** community violence, violence, adolescent, mental health, depression, post-traumatic stress, anxiety, internalizing symptoms

## Abstract

**Purposes:**

Mental disorders are responsible for 16% of the global burden of disease in adolescents. This review focuses on one contextual factor called community violence that can contribute to the development of mental disorders

**Objective:**

To evaluate the impact of community violence on internalizing mental health symptoms in adolescents, to investigate whether different proximity to community violence (witness or victim) is associated with different risks and to identify whether gender, age, and race moderate this association.

**Methods:**

systematic review of observational studies. The population includes adolescents (10-24 years), exposition involves individuals exposed to community violence and outcomes consist of internalizing mental health symptoms. Selection, extraction and quality assessment were performed independently by two researchers.

**Results:**

A total of 2987 works were identified; after selection and extraction, 42 works remained. Higher exposure to community violence was positively associated with internalizing mental health symptoms. Being a witnessing is less harmful for mental health than being a victim. Age and race did not appear in the results as modifiers, but male gender and family support appear to be protective factors in some studies.

**Conclusion:**

This review confirms the positive relationship between community violence and internalizing mental health symptoms in adolescents and provides relevant information that can direct public efforts to build policies in the prevention of both problems.

**Supplementary Information:**

The online version contains supplementary material available at 10.1186/s12888-022-03873-8.

## Background

Mental disorders account for 16% of the global disease burden in adolescents. The onset of half of all cases of mental disorders occurs by the age of 14 years, and the onset of 75% of all cases occurs by the mid-20s [1]. Adolescence is a moment of considerable physical, psychological, cognitive, and sociocultural changes and an expected period of crisis [2]. The natural transition from childhood to adult life could mask some mental health symptoms. Most mental disorders go undetected, dragging their consequences to adulthood and causing functional impairment [1].

Mental health problems can be divided into externalizing and internalizing behaviour problems [3]. The first group is characterized by behaviours that target the environment and others. In internalizing problems, behaviours target the individual, including common mental disorders and post-traumatic stress disorder.

Common mental disorders correspond to a group of symptoms, including anxiety, depression, and somatic complaints, but not necessarily a pathology; common mental disorders are highly prevalent [4]. A systematic review estimated the prevalence of past-year and lifelong common mental disorders worldwide as 17.6% and 29.2%, respectively [5]. A study conducted in Brazil with adolescents showed a prevalence of common mental disorders of 30.0% [6]. Post-traumatic stress disorder is also a significant health condition that affects children and adolescents. It consists of the presence of intrusive thoughts relating to a traumatic event, avoidance of reminders of the trauma, hyperarousal symptoms, and negative alterations in cognitions and mood [7]. A meta-analysis showed that the overall rate of post-traumatic stress disorder in this group was 15.9% (95% CI 11.5–21.5) [8]. Another meta-analysis that focused on delayed post-traumatic stress disorder found that the proportion of post-traumatic stress disorder cases with delayed post-traumatic stress disorder was 24.8% (95% CI = 22.6% to 27.2%) [9].

Understanding the determinants of mental disorders is not an easy task, since these disorders are considered multifactorial phenomena. The literature has pointed out that genetic characteristics, the history of child development, and contextual factors are the main drivers of the development of mental illness among adolescents [10]. Among contextual factors, those considered the most important are low socioeconomic level, family conflicts and victimization of different forms of violence [11]. Adolescents can be especially vulnerable to community violence and its consequences [12]. At this stage, youths' circulation outside the home and without their families will increase [13]. Inexperience, emotional immaturity and the need to test limits, combined with this increase in community space circulation, could lead to exposure to violence and maximize its mental health effects. The increase in community violence in recent years is a global problem, and such violence is most frequent in low- and medium-income countries [14].

This review will focus on one contextual factor influencing mental disorders in adolescence: community violence [15, 16]. Community violence is a type of interpersonal violence that occurs among individuals outside of personal relationships. It includes acts that occur in the streets or within institutions (schools and workplaces) [17]. In addition, community violence can be experienced directly (victimization) or indirectly (witnesses and hearing about).

Estimating the impact of exposure to community violence on adolescents’ mental health has been at the core of a large body of research. Two previous meta-analyses showed a mild to moderate and positive effect of community violence on adolescents’ mental health [18, 19]. However, these associations need to be confirmed since many primary studies were published after 2009. Additionally, there are still significant gaps to be addressed. For instance, it is not clear whether different degrees of proximity to community violence (victimization, witnessing, or hearing about) influence mental health outcomes (depression, anxiety, and post-traumatic stress disorder) at different magnitudes. Moreover, it is not clear whether gender, race and age can moderate this relationship, as well as other factors such as family constitution and interpersonal relations.

This review's main objective is to systematize the scientific literature that has estimated the impact of community violence on adolescents' mental health. Other goals are (i) to investigate whether different proximity to community violence is associated with different magnitudes of common mental disorders or post-traumatic stress disorder and (ii) to identify whether gender, age, and race moderate the association between community violence and internalizing symptoms.

## Methodology

All methods were carried out in accordance with the *Preferred Reporting Items for Systematic Reviews and Meta-analysis Protocols (PRISMA-P) 2015 checklist* and *Joanna Briggs Institute Reviewers Manual—Chapter 7: Systematic reviews of aetiology and risk* [20, 21]. The protocol is registered in the International Prospective Register of Systematic Reviews (PROSPERO) – CRD 42019124740.

The review question was: 'Are adolescents exposed to higher levels of community violence at higher risk of developing internalizing mental health symptoms?'

### Eligibility criteria

#### Population

Following the World Health Organization (WHO) classification for adolescence, studies were selected if adolescents in the sample were aged 10 to 24 years. To be included in the review, adolescents participating in the studies needed to be in this age group at the time of outcome measurement [22]. There were no exclusion criteria.

#### Exposure of interest

Our exposure of interest is community violence. Community violence events that occurred inside institutions, such as schools and workplaces, and events of sexual nature, such as rape or other types of sexual aggression, were excluded. This choice was based on the fact that these types of community violence have different effects and magnitudes on adolescent mental health [23–26].

The inclusion criteria were original studies measuring community violence through questionnaires (answered by adolescents, parents, relatives or professionals responsible for the child and teachers) or crime rates. The exclusion criteria were original studies that included other types of violence, such as domestic violence, bullying, or sexual violence, that could not be separated from community violence. Comparison groups included adolescents not exposed or exposed to community violence at a lower level. There were no exclusion criteria for comparison.

#### Outcomes/dependent variables

This review considered studies that included internalizing symptoms as the primary outcome, represented by post-traumatic stress disorder, common mental disorder symptoms, depression, and anxiety. As inclusion criteria, studies that measured mental health symptoms through a questionnaire with the adolescents themselves, their parents, teachers, or professionals related to them and that had an association measure for the outcome were used. Exclusion criteria were applied for studies with association measures from regression models without adjustment.

#### Study design

This review included the following study designs: longitudinal, cross-sectional, and case–control. Case reports, case series, reviews, qualitative methodologies, interventions, descriptive studies, and methodologic studies were excluded.

### Information sources

The search was performed in six allied health research databases: Medline (accessed through PubMed), PsycINFO, Embase, LILACS (*Literatura Latino-americana e do Caribe em Ciências da Saúde*), Web of Science, and Scopus. Regarding grey literature, only those corresponding to theses and dissertations were included. These were identified in the databases above, and “ProQuest Dissertation and Theses” was used to search for full texts. The search was conducted on February 5^th^, 2019, and updated in January 14^th^, 2021 and no filters for years of publication or language were applied. After the third phase of selection, all studies included in the review had their reference lists analysed by two independent researchers to search for additional works.

### Search strategy

**S**earch terms

were based on the review question and were constructed with a librarian (APPENDIX I). The main concepts were as follows: "adolescents" OR "youth" OR "teenagers" AND "community violence" OR "urban violence" OR "neighborhood violence" AND "mental health" OR "anxiety" OR "depression" OR "post-traumatic" OR "internalizing" OR "psychological symptoms". A librarian worked on obtaining the full-text works, seeking bibliographic bases, libraries, and contact authors.

### Study selection

Data selection was carried out in three stages: title, abstract and full texts. During all phases, two researchers performed critical readings to apply the pre-established inclusion and exclusion criteria. All stages were preceded by a pilot that included 10% of the total number of works in each phase (concordance rate 80-97%). In the first and second stages of selection, any disagreements were included. At the second stage of selection, we decided to exclude externalizing outcomes. In the third stage, we discussed all the discrepancies. When there were discrepancies, a third researcher was called. All reasons for exclusion were registered.

The authors of five studies were contacted for clarification. The corresponding authors of each work in which queries arose during the selection phase were contacted by e-mail. In cases where we did not receive a response, a new e-mail was sent 15 days later. The queries referred to the presence of questions about sexual and school violence in the violence questionnaires and a lack of reported confidence intervals (CIs) in the studies.

### Data extraction

Data were extracted using EpiData 3.1 with a standardized formulary tested in the pilot. Extracted information included the following: (i) study design, setting, times of measures and recruitment; (ii) demographic population; (iii) exposure characteristics – classification subtypes and measurement instrument; (iv) comparison group; (v) outcomes – types and measurement instruments; and (vi) association measures. Again, two review researchers worked independently. All papers included at this phase were discussed. In two studies, a third researcher was consulted to decide about discrepancies. At the end of the extraction phase, 42 studies were divided into two groups: 21 studies with complete information that were included in the meta-analysis and 21 studies with incomplete information included only in the qualitative synthesis.

### Assessment of methodological quality

The quality of the studies was also evaluated independently by two researchers. The formulas used were adaptations, also tested in the pilot phase, from a predefined quality assessment form for cohort/case–control studies and descriptive studies published in the Joanna Briggs Institute Reviewers' Manual [27]. Studies were classified into three categories: low, intermediate, and high quality. Researchers defined cut-off points; all questions had the same weight in the final punctuation. Discrepancies were discussed, and a consensus was achieved in all cases. Critical appraisal tools are presented in APPENDIX IV.

### Synthesis of the results

Results are presented in qualitative synthesis. A subgroup of 21 studies underwent quantitative synthesis. Forest plots were displayed to visualize the results. Heterogeneity was evaluated by the I^2^ test, which describes the proportion of variation across the studies due not to chance but rather to heterogeneity [28, 29]. The higher the percentage, the higher the level of heterogeneity. Because heterogeneity was still high when adopting the random effect model, reasons for these were investigated, and subgroup analysis was conducted – stratification by proximity to community violence (witness and victim) and types of outcomes (post-traumatic stress disorder, depression and internalizing symptoms) were performed. Because heterogeneity was still high in almost all forest plots, it was not possible to construct funnel plots to evaluate possible publication bias. We report our findings in accordance with PRISMA guidelines [30].

## Results

**Fig. 1:**
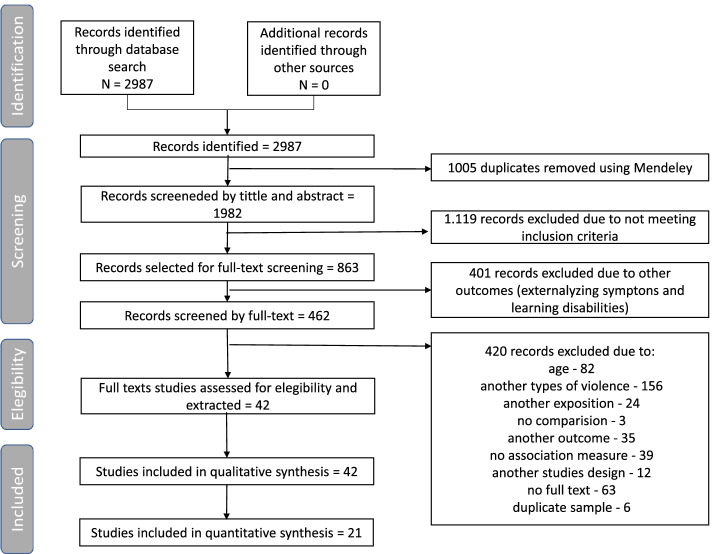
Flowchart of the selection and extraction phase

After a search in databases, 2987 works were identified, and no additional papers were found through other sources. Of these quantities, 1005 duplicates were removed, and the selection phase started with 1982 records. During stages 1 and 2 of selection, 1.119 records were excluded, leaving 863 for the third phase. The eligibility phase started with 42 works. Of these, 21 were included in the quantitative synthesis. Details are presented in Fig. 1.

The results are presented in the following manner: 42 studies included in qualitative syntheses had their main characteristics presented in Table 1, and their results were described according to the review objectives. Quality assessments are presented in Table 2 and 3; 30 were considered high quality, 11 intermediate and one low quality.

**Table 1:** Characteristics, outcomes and association measures of the included studies

Authors and year of publication	Country and city	Sample characteristics (number and age [years] of participants)	Types of community violence exposure (CVE) and outcome	Association measure, confidence interval or standard error, P value
**Longitudinal studies**				
Daviera et al., 2019 [31]	Country: USA City: Chicago	N = 314 • Age Average 16.17 (SD 0.79)	• Crime statistics • Subtypes: Trait anxiety	Pervasive CVE: • Home-based × school-based CVE × anxiety: β = -0.69 (SE 0.33)* Spatially proximate CVE: • Most distal CVE × anxiety: β = -0.01 (SE 0.05) • Most proximal CVE × anxiety: β = -0.19 (SE 0.21) Chronic CVE: • Long-term CVE × anxiety: β = -0.017 (SE 0.064) • Mid-term CVE × anxiety: β = -0.031 (SE 0.070) • Short-term CVE × anxiety: β = -0.066 (SE 0.044)
Elsaesser et al., 2018 [32]	Country: USA City: Chicago	N = 246 • Mean age 14.8 (SD 1.2)	• Classified into victim and witness • Subtypes: Depression	• Victim of CVE × depression (longitudinal): β = -0.03 (SE NI) * • Witness of CVE × depression (longitudinal): β = 0.14 (SE NI) • Victim of CVE × depression (cross-sectional): β = 0.14 (SE NI) * • Witness of CVE × depression (cross-sectional): β = 0.08 (SE NI)
Eisman et al., 2015 [33]	Country: USA City: Flint	N = 824 • Mean age 14.86 (SD 0.64)	• Not classified into victim and witness • Subtypes: Depressive symptoms	• CVE × depressive symptoms: β = 0.03 (SE 0.02) *
Farrell et al., 1997 [34]	Country: USA City: Richmond	N = 436 • Mean age 11.7 (SD 0.6)	• Not classified into victim and witness • Subtypes: Emotional distress	• CVE × emotional distress (male): β = 0 (SE NI) • CVE × emotional distress (female): β = 0.07 (SE NI)
Gepty et al., 2019 [35]	Country: USA City: Philadelphia	N = 309 • Age Average 12.9 (SD 0.61)	• Crime statistics • Subtypes: Depression	• CVE × rumination × depression: β = 0.0004 (SE NI) * • No CVE × rumination × depression: β = -0.0001 (SE NI) • Violent crime × rumination × depressive symptoms: β =0.0002 (SE 0.00)* •Nonviolent crime × rumination × depressive symptoms: β =0.00 (SE 0.00)
Kohl et al., 2015 [36]	Country: USA City: Chicago	N = 320 • Mean age 11.65 (SD 0.7)	• Not classified into victim and witness • Subtypes: Depression	• CVE 6th grade × Depression 7th grade: β = 0.15 (SE NI) * • CVE 7th grade × Depression 8th grade: β = 0.21 (SE NI) *
Authors and year of publication	**Country and city**	**Sample characteristics (number and age [years] of participants)**	**Types of CV exposure and outcome**	**Association measure, confidence interval or standard error, P value**
Lambert et al., 2008 [37]	Country: USA	N = 473 • Mean age 11.77 (SD 0.35)	• Not classified into victim and witness • Subtypes: Depression	• CVE × depression (male): β = 0.20 (SE NI) * • CVE × depression (female): β =0.20 (SE NI) *
Latzman et al., 2005 [38]	Country: USA	N = 8939 • Mean age 15.18 (SD 1.6)	• Not classified into victim and witness • Subtypes: Depression	• CVE × depression: β = 0.02 (SE 0.03) *
McKelvey et al., 2015 [39]	Country: USA City: Boston	N = 473 • Mean age 18 (SD NI)	• Not classified into victim and witness • Subtypes: Internalizing symptoms	• CVE × internalizing symptoms: β = 0.02 (SE 0.01) • CVE × Gender × internalizing symptoms: β = -0.03 (SE 0.01) • CVE × Gender × cohesion × internalizing symptoms: β = 0.11 (SE 0.01) *
Mrug et al., 2010 [40]	Country: USA City: Birmingham	N = 603 • Mean age 13.20 (SD 0.9)	• Classified into victim and witness • Subtypes: Depression, Anxiety	• Victim of CVE × depression: β = 0.01 (SE NI) • Witness of CVE × depression: β = 0.6 (SE NI) • Victim of CVE × anxiety: β = 0.01 (SE NI) • Witness community × anxiety: β = 0.06 (SE NI)
Ozer et al., 2005 [41]	Country: USA	N = 73 • Age not specified numerically (7th-8th grade)	• Not classified into victim and witness • Subtypes: Depression, Anxiety, Intrusive symptoms	• Exposure to CVE × anxiety: β = 0.31 (SE NI)* • Exposure to CVE × intrusive symptoms: β = 0.34 (SE NI)* • Exposure to CVE × depression: β = 0.12 (SE NI)
Sun et al., 2020 [42]	Country: USA	N = 85 • Mean age 15.55 (SD 1)	• Not classified into victim and witness • Subtypes: PTSD	• CVE × PTSD: β = 0.34 (CI 0.16-0.52)* • CVE × emotional regulation (internally dysfunctional): β = 0.2 (CI 0.06–0.35)* • CVE × emotional regulation (externally functional): β = 0.05 (CI -0.12–0.22) • CVE × emotional regulation (internally functional): β = 0.09 (CI -0.05–0.24) • CVE × emotional regulation (externally dysfunctional): β = 0.11 (CI -0.06–0.27)
**Cross-sectional study**				
Aisenberg et al., 2008 [43]	Country: USA City: Los Angeles	N = 137 • Mean age 12.41 (SD 0.79)	• Not classified into victim and witness • Subtypes: PTSD	• CVE × PTSD: β = 4.41 (SE 2.81)
Bacchini et al., 2011 [44]	Country: Italy City: Naples	N = 489 • Mean age 17.53 (SD 1.24)	• Classified into victim and witness • Subtypes: Anxiety, Depression	• Victim of CVE × anxiety/depression: β = 0.25 (SE NI) * • Victim of CVE × gender × anxiety/depression: β = 0.15 (SE NI) * • Witness of CVE × anxiety/depression: β = 0.8 (SE NI) • Witness of CVE × parental monitoring × anxiety/depression: β = -0.13 (SE NI) *
Authors and year of publication	**Country and city**	**Sample characteristics (number and age [years] of participants)**	**Types of CV exposure and outcome**	**Association measure, confidence interval or standard error, P value**
Campo-Ríos et al., 2020 [45]	Country: Mexico City: Ciudad Juárez	N = 298 • Mean age 19.28 (SD 0.5)	• Contextual violence in neighbourhood (heard about). Victim and witness not measured • Subtypes: PTSD	• CVE × PTSD × female: β = 0.14 (SE NI)* • CVE × PTSD × male: β = 0.12 (SE NI)* • CVE × PTSD: β = 0.14 (SE NI)*
Cecil et al., 2014 [46]	Country: England City: London	N = 204 • Mean age 18.85 (SD 2.27)	• Not classified into victim and witness • Subtypes: PTSD Internalizing symptoms	• CVE × internalizing problems: β = 0.01 (CI -0.21-0.56) • CVE × PTSD: β = 0.10 (CI 0.04-0.17) *
Chen et al., 2020 [47]	Country: USA City: Chicago	N = 2621 • Age Average 12.47 (SD 0.98)	• Not classified into victim and witness • Subtypes: Depression	• Latinx × CVE × depression: β = 0.48 (SE 0.07)* • Black × CVE × depression: β = 0.50 (SE 0.08)* • White × CVE × depression: β = 0.77 (SE 0.06)*
Cuartas et al., 2019 [48]	Country: Colombia City: Bogotá	N = 300 • Mean age 14.52 (SD 1.72)	• Crime statistics • Subtypes: Common mental disorders PTSD	• CVE (homicide rates) × common mental disorders β = 0.17 (SE 0.06)* • CVE (homicide rate) × perceived the neighbourhood as unsafe × common mental disorders: β = 0.23 (SE 0.07)* • CVE (homicide rate) × perceived the neighbourhood as safe × common mental disorders: β = -0.24 (0.13) • CVE (homicide rates) × PTSD: β = 0.14 (SE 0.06)* • CVE (homicide rates) × poverty × PTSD: β = 0.55 (SE 0.13)* • CVE (homicide rates) × victim of crime × PTSD: β = 0.45 (SE 0.12)* • CVE (homicide rates) × perceived neighbourhood as unsafe × PTSD: β = 0.20 (SE 0.07)* • CVE (homicide rates) × perceived neighbourhood as safe × PTSD: β = -0.19 (SE 0.10)*
Darawshy et al., 2018 [49]	Country: Israel	N = 760 • Mean age 16.1 (SD 1.27)	• Not classified into victim and witness • Subtypes: Internalizing symptoms	• CVE × internalizing symptoms: β = 1.74 (SE 0.28) *
Authors and year of publication	**Country and city**	**Sample characteristics (number and age [years] of participants)**	**Types of CV exposure and outcome**	**Association measure, confidence interval or standard error, P value**
Donernberg et al., 2020 [50]	Country: South Africa	N = 120 • Age Average 14.39 (SD 1.82)	• Not classified into victim and witness • Subtypes: Internalizing symptoms	• Witness of CVE × internalizing symptoms × girls: β = -0.06 (SE 0.14) • Witness of CVE × internalizing symptoms × boys: β = -0.03 (SE 0.14)
Ford et al., 2010 [51]	Country: USA	N = 4023 •Age 12-17 years (SD NI)	• Classified into victim and witness • Subtypes: Depression PTSD	• Victim of CVE × PTSD: OR = 5.58 (CI 3.29–9.45) * • Victim of CVE × depression: OR = 2.61 (CI 1.64–4.16) * • Witness of CVE × PTSD: OR = 3.64 (CI 2.35–5.64) * • Witness of CVE × depression: OR = 2.55 (CI 1.80–3.61) *
Foster et al., 2004 [52]	Country: USA	N = 146 • Mean age 13.16 (SD 1.01)	• Classified into victim and witness • Subtypes: Depression Anxiety PTSD	• Victim of CVE × anxiety: β = 0.31 (SE NI) * • Victim of CVE × depression: β = 0.14 (SE NI) * • Victim of CVE × PTSD: β = 0.33 (SE NI) *
Goldman-Mellor et al., 2016 [53]	Country: USA	N = 4462. • Mean age 14.6 (SD 0.22)	• Crime statistics • Subtype: Psychological distress/depression and anxiety symptoms	• Exposure to CVE × psychological distress/depression and anxiety symptoms: OR = 1.41 (CI 0.6–3.32)
Grinshteyn et al., 2018 [54]	Country: USA	N = 5519 • Maximum age 18, minimum age 11	• Classified into victim and witness • Crime statistics • Subtypes: Depressive symptoms Internalizing symptoms	• Victim of CVE (violent crime) × depressive symptoms: β = 5.13 (SE NI)* • Seeing/knowing victim of CV (violent crime) × depressive symptoms: β = 3.46 (SE NI) * • Witness of CVE (violent crime) × depressive symptoms: β = 2.09 (SE NI)* • Increased of 1 crime/1000 people × depressive symptoms: β = 0.06 (SE NI) *
Haj-Yahia et al., 2021 [55]	Country: Israel City: Jerusalem	N = 1930 • Mean age 16.36 (SD 1.04)	• Not classified into victim and witness • Subtypes: Internalized problems	• Witness of CVE × internalizing problems: β = 0.17 (SE NI)* • Victmim of CVE × internalizing problems: β = -0.04 (SE NI) • Witness of CV × female gender × internalizing problems: β = -0.09 (SE NI) • Victim of CVE × female gender × internalizing problems: β = 0.25 (SE NI)* • Witness of CVE × family support × internalizing problems: β = 0 (SE NI) • VCV × family support × internalizing problems: β = 0 (SE NI)
Henry et al., 2015 [56]	Country: USA	N = 106 • Mean age 15.41 (14-17)	• Not classified into victim and witness • Subtypes: Depressive symptoms	• CVE × depressive symptoms: β = 0.23 (SE 0.07) * • CVE × cultural appreciation of legacy × depressive symptoms: β = -0.11 (SE 0.3) *
Authors and year of publication	**Country and city**	**Sample characteristics (number and age [years] of participants)**	**Types of CV exposure and outcome**	**Association measure, confidence interval or standard error, P value**
Ho et al., 2010 [57]	Country: China City: Hong Kong	N = 405 • Mean age 13.59 (SD 1.43)	• Classified into victim and witness • Subtypes: Emotional problems	• Witness of CVE × emotional problems: β = -0.09 (SE NI) • Victim of CVE × emotional problems: β = 0.17 (SE NI) *
Howard et al., 2010 [58]	Country: USA	N = 174 • Age: 9th grade	• Not classified into victim and witness • Subtypes: Anxiety/Stress	• CVE × distress symptoms: β = 0.35 (SE NI) * • CVE × family support × distress symptoms: β = -0.27 (SE NI)*
Kaminer et al., 2013 [59]	Country: South Africa City: Cape Town	N = 230 • Mean age 17.45 (SD 1.59)	• Classified into victim and witness • Subtypes: PTSD	• Victim of CVE × PTSD: β = 0.77 (SE 0.28) * • Victim of CVE × PTSD (girls): β = 0.88 (SE 0.33) * • Witness of CVE × PTSD: β = 0.13 (SE 0.12) • Witness of CVE × PTSD (girls): β = 0.04 (SE 0.15)
Klodnick et al., 2014 [60]	Country: Israel	N = 1571 • Mean age 16.26 (SD 1.05)	• Classified into victim and witness • Subtypes: PTSD	• Victim of CVE × PTSD: β = 4.91 (SE 0.49) * • Witness of CVE × PTSD: β = 1.86 (SE 0.28) * • Ethnicity × CVE × PTSD: β = -0.99 (SE 0.85)
Lätsch et al., 2016 [61]	Country: Switzerland	N = 6749 • Mean age 15.5 (14-17)	• Not classified into victim and witness • Subtypes: Emotional symptoms	• CVE × emotional symptoms: β = 0.16 (SE NI) *
Leblanc et al., 2011 [26]	Country: USA	N = 90 • Mean age 16.4 (SD 1.37)	• Not classified into victim and witness • Subtypes: Psychological distress	• CVE × Psychological distress: β = 0.02 (SE NI) • CVE × Problem solving ability of adolescents × Psychological distress: β = 0.43 (SE NI)*
Mendelson et al., 2010 [62]	Country: USA City: Baltimore	N = 629 • Mean age 18.7 (SD 1.77)	• Not classified into victim and witness • Subtype: Depressive symptoms	• CVE × depressive symptoms: OR= 2.27 (CI 1.57–3.29)*
O’Donnell et al., 2011 [63]	Country: Republic of the Gambia	N = 653 • Mean age 17.76 (SD 1.53)	• Classified into victim and witness • Subtypes: TEPT	• Witness of CVE × PTSD: β = 0.18 (SE 0.10)* • Witness of CVE × school climate × PTSD: β = -0.10 (SE 0.04)* • Victim of CVE × PTSD: β = 0.12 (SE 0.13) * • Victim of CVE × school climate × PTSD: β = 0.12 (SE 0.5)*
Authors and year of publication	**Country and city**	**Sample characteristics (number and age [years] of participants)**	**Types of CV exposure and outcome**	**Association measure, confidence interval or standard error, P value**
O’Leary et al., 2020 [64]	Country: USA	N = 196 • Mean age 12.41 (SD 1.14)	• Not classified into victim and witness • Subtypes: PTSD depression	• CVE × PTSD: β = 0.4 (SE 0.07)* • CVE × cognitive reappraisal × PTSD: β = -0.1 (SE 0.1) • CVE × expressive suppression × PTSD: β = 0.4 (SE 0.07) • CVE × depression: β = 0.33 (SE 0.04)* • CVE × cognitive reappraisal × depression: β = -0.08 (SE 0.05) • CVE × expressive suppression × depression: β = 0.14 (SE 0.04)* •Gender was tested as modifier and was not significant.
Ozer et al., 2004 [65]	Country: USA City: California	N = 349 • Median age 12 (SD NI)	• Not classified into victim and witness • Subtypes: Depressive symptoms PTSD	• CVE × depressive symptoms: β = 0.11 (SE NI)* • CVE × PTSD: β = 0.19 (SE NI)*
Plessis et al., 2015 [66]	Country: South Africa City: Cape Town	N = 616 • Mean age 12.8 (SD 0.74)	• Classified into victim and witness • Subtypes: Depression	•Witness of CVE × depression: β = 0.02 (SE 0.04) • Victimof CVE × depression: β = 0.07 (SE 0.14)
Sargent et al., 2019 [67]	Country: USA	N = 403 • Mean age 12.63 (SD 0.99)	• Classified into victim and witness • Subtypes: Depression PTSD Anxiety	• Victim of CVE × depression: β = 0.49 (SE NI)* • Witness of CVE × depression: β = 0.31 (SE NI)* • Victim of CVE × anxiety: β = 0.54 (SE NI)* • Witness of CVE × anxiety: β = 0.50 (SE NI)* • Victim of CVE × PTSD: β = 0.61 (SE NI)* • Witness of CVE × PTSD: β = 0.53 (SE NI)*
Shukla et al., 2015 [68]	Country: USA	N = 233 • Mean age 17 (SD 0.62)	• Classified into victim and witness • Subtype: Depression	• Witness of CVE × depression: β = 0.03 (SE 0.15) • Victim of CVE × depression: β = -0.04 (SE 0.27)
Boney-McCoy et al., 1995 [69]	Country: USA	N = 2000 • Mean age 8 (10-16)	• Not classified into victim and witness • Subtypes: PTSD Sadness	• Aggravated assault × PTSD (female): β = 0.42 (SE 2.81) * • Simple assault × PTSD (female): β = 0.20 (SE NI) • Attempted kidnapping × PTSD (female): β = 0.51 (SE NI)* • Aggravated assault × PTSD (male): β = 0.36 (SE NI)* • Simple assault × PTSD (male): β = 0.12 (SE NI) • Attempted kidnapping × PTSD (male): Β = 0.035 (SE NI)*
Sui et al., 2018 [70]	Country: South Africa	N = 1574 • Mean age = 16 (12-22)	• Classified into victim and witness • Subtype: Depression	• Witness of CVE × depression: β = 0.03 (SE 0.15) • Victim of CVE × depression: β = -0.04 (SE 0.27)
Authors and year of publication	**Country and city**	**Sample characteristics (number and age [years] of participants)**	**Types of CV exposure and outcome**	**Association measure, confidence interval or standard error, P value**
Velez-Gomez et al., 2013 [71]	Country: Colombia City: Medellín	N = 289 • Mean age 10.17 (SD 1.25)	• Crime statistics • Subtypes: Negative mood, Interpersonal problems, Ineffectiveness, Negative self-esteem	• CVE × ineffectiveness: β = 0.21 (SE NI) *

Abbreviations: USA: United States of America; SD = standard deviation; β: beta; OR: odds ratio; CVE: community violence exposure; PTSD: post-traumatic stress disorder; SE: standard error; NI: no information; *: p < 0.05.

**Table 2:** Quality assessment of the included cohort studies

Study	Sample	Comparable groups	Selection bias	Confounders	Follow-up	Losses	Outcome measurable	Statistical analysis	Exposure measurable	Score
**Intermediate quality**										
Farrell et al. (1997)	N	U	N	N	Y	N	Y	Y	Y	18
Grinshteyn et al. (2018)	U	U	N	Y	Y	N	Y	Y	N	19
**High quality**										
Sun et al. (2020)	N	Y	U	N	Y	U	Y	N	N	20
Latzman et al. (2005)	Y	Y	U	Y	Y	Y	N	Y	N	20
Eisman et al. (2015)	Y	U	N	Y	Y	Y	Y	Y	N	22
McKelvey et al. (2015)	Y	U	N	Y	Y	Y	Y	Y	N	22
Gepty et al, (2019)	U	Y	U	Y	Y	Y	Y	Y	N	23
Lambert et al. (2008)	Y	Y	U	Y	Y	N	Y	Y	Y	24
Kohl et al. (2015)	U	Y	U	Y	Y	N	Y	Y	Y	26
Elsaesser et al. (2018)	Y	Y	Y	Y	Y	Y	Y	Y	Y	27
Mrug et al. (2010)	Y	Y	Y	Y	Y	Y	Y	Y	U	27
Ozer et al. (2005)	Y	Y	Y	Y	Y	Y	Y	Y	Y	27
Davieira et al. (2019)	Y	Y	Y	Y	Y	Y	Y	Y	Y	27

**Table 3:** Quality assessment of the included cross-sectional studies

Study	Randomized sample	Sample definition	Confounders	Comparable groups	Losses	Outcome measurable	Statistical analysis	Exposure measurable	Score
**Low quality**									
Aisenberg et al. (2008)	N	N	N	N	N	Y	N	Y	12
**Intermediate quality**									
Kaminer et al. (2013)	N	Y	N	N	N	U	Y	U	14
Ozer et al. (2004)	N	Y	N	N	N	Y	N	Y	14
Shukla et al. (2015)	N	N	Y	N	N	U	Y	U	14
Leblanc et al. (2011)	N	N	Y	N	N	Y	Y	Y	16
Henry et al. (2015)	N	Y	Y	N	Y	Y	Y	Y	17
O’Donnell et al. (2001)	N	Y	Y	N	U	U	Y	U	17
Mendelson et al. (2010)	N	Y	Y	N	Y	Y	Y	N	18
**High quality**									
Ford et al. (2010)	Y	Y	N	N	Y	Y	Y	U	19
Chen et al. (2020)	U	Y	Y	U	U	Y	Y	N	19
Campo-Ríos et al. (2020)	U	U	N	N	N	Y	Y	Y	19
Donernberg et al. (2020)	U	N	N	U	N	Y	Y	Y	19
Howard et al. (2010)	N	Y	Y	N	U	Y	Y	Y	19
Foster et al. (2004)	N	Y	Y	N	Y	Y	Y	Y	20
Ho et al. (2010)	N	Y	Y	N	Y	Y	Y	Y	20
Klodnick et al. (2014)	N	Y	Y	Y	Y	U	Y	U	20
Sui et al. (2018)	Y	Y	Y	U	N	Y	Y	U	20
Leary et al. 2021	U	Y	Y	N	U	Y	Y	Y	20
Sargent et al. 2021	U	U	Y	U	U	Y	Y	N	21
Bacchini et al. (2011)	N	Y	Y	N	Y	Y	Y	Y	22
Darawshy et al. (2018)	N	Y	Y	Y	N	Y	Y	Y	22
Boney-McCoy et al. (1995)	Y	Y	Y	N	N	Y	Y	Y	22
Plessis et al. (2015)	N	Y	Y	Y	Y	Y	Y	Y	22
Haj-Yahia et al. (2021)	Y	Y	Y	N	Y	Y	Y	Y	22
Cecil et al. (2014)	N	Y	Y	N	Y	Y	Y	Y	23
Velez-Gomez et al. (2013)	Y	Y	Y	Y	Y	Y	Y	Y	24
Goldman-Mellor et al. (2016)	Y	Y	Y	Y	Y	Y	Y	Y	24
Lätsch et al. (2016)	Y	Y	Y	Y	Y	Y	Y	Y	24
Cuartas et al. (2019)	Y	Y	Y	Y	Y	Y	Y	Y	24

Answers: Y – Yes, N – No, U – Undefined. Score: N (1 point); U (2 points); Y (3 points). The studies were ordered according to their quality. Light grey colour – low quality; medium grey – intermediate quality; dark grey – high quality. The work by Grinshteyn et al. (2018) was evaluated as a longitudinal study because of its study design, but the results presented in Table 1 are classified as cross-sectional because they were statistically analysed using the procedures for cross-sectional studies.

A subgroup of 21 studies could be meta-analysed. The first forest plots were generated and included all 21 studies. For these, we worked with the concept of general community violence and only one type of outcome, so for the studies that had more than one association measure (for victim and witnessing, for example), a weighted average was calculated, and the same was done for the studies that had more than one outcome. The I^2^ value was 53.8%, with a p value of 0.003, thus indicating substantial heterogeneity [72]. Subgroup analysis was conducted with stratification by proximity of community violence (CV) (witness and victim) and then with types of outcomes (post-traumatic stress disorder, depression and internalizing symptoms). The only graphics presented were those with heterogeneity smaller than 60%, which corresponds to the subgroups of post-traumatic stress disorder and internalizing symptoms as outcomes.

**Fig. 2:**
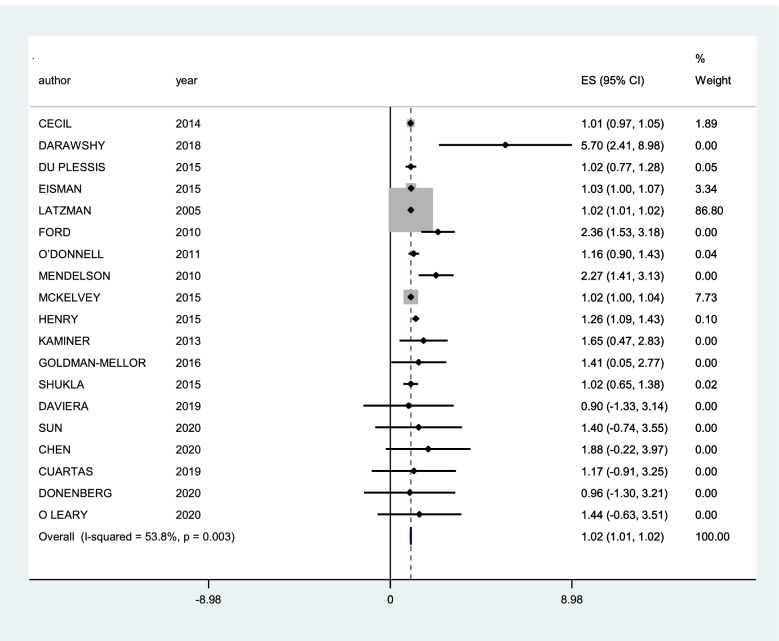
Forest plot of studies with general community violence as exposure and any type of internalizing mental disorders as outcomes

**Fig. 3:**
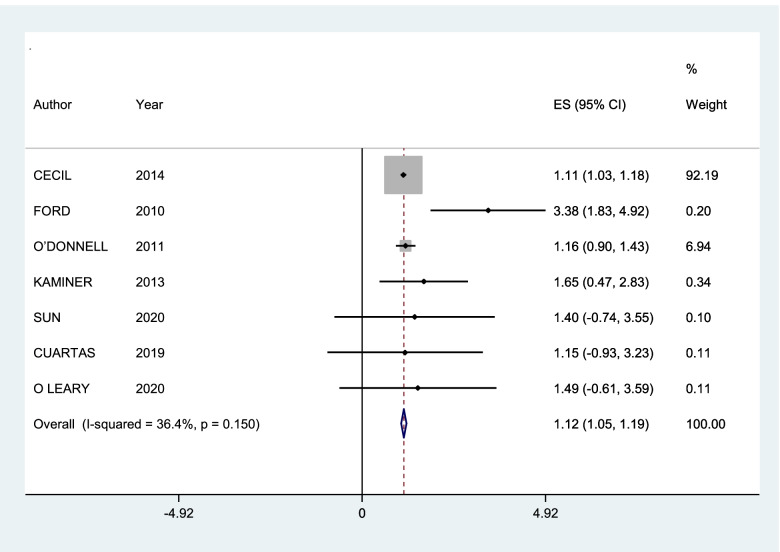
Forest plot of subgroups of studies that considered post-traumatic stress disorder as an outcome

**Fig. 4:**
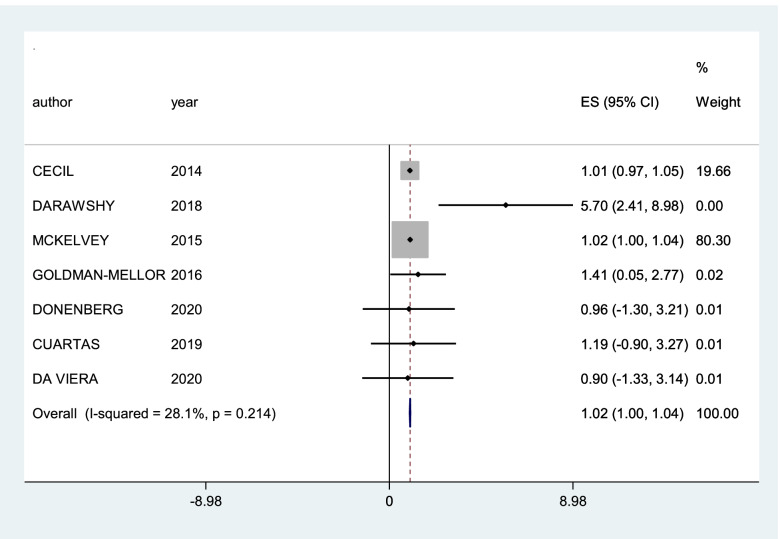
Forest plot of subgroups of studies that considered internalizing symptoms as outcomes

The results of the summary measures must be interpreted with caution. Only some of the qualitative synthesis studies presented complete data that would allow inclusion in the quantitative synthesis. The first graph generated (Fig. 2) shows high heterogeneity, and the graphs presented for the outcomes of post-traumatic stress disorder (Fig. 3) and internalizing symptoms (Fig. 4) do not show high heterogeneity but represent a small group of studies compared to the total number included in the review. Nevertheless, it was possible to see a small but statistically significant greater effect for post-traumatic stress disorder than internalizing symptoms.

Legend – Answers: Y – Yes, N – No, U – Undefined. Score: N (1 point); U (2 points); Y (3 points). The studies were ordered according to their quality. Light grey colour – low quality; medium grey – intermediate quality; dark grey – high quality. The work by Grinshteyn et al. (2018) was evaluated as a longitudinal study because of its study design, but the results presented in Table 1 are classified as cross-sectional because they were statistically analysed using the procedures for cross-sectional studies.

### Mental health symptoms and exposure to community violence

Twenty-eight studies did not consider different degrees of proximity to violence in their analysis [26, 31–43, 45–50, 53, 55, 56, 58, 61, 62, 64, 65, 69, 71, 73]. Of these, twenty-three found a significant association between exposure and outcome (Table 1).

Five studies did not find community violence to be a risk factor for internalizing mental health symptoms [26, 34, 43, 53]. Le Blanc et al. [26] justified the lack of association between community violence and outcomes analysed by the fact that other types of violence (home and school) were considered in the statistical analysis and could have influenced the results for a null association. Farrel et al. [34] discussed their results in light of the desensitization hypothesis since the sample has a high prevalence of community violence [74–76]. Goldman-Mellor et al. [53] compared their sample perception of violence and objectively measured neighbourhood violence derived from criminal statistics. Perception of violence in the neighbourhood is a different concept than exposure to community violence because the first is related to how adolescents see the environment in which they live. The authors found that adolescents who perceived their neighbourhood unsafe had a nearly 2.5-fold greater risk of psychological distress than those who believed their neighbourhood was safe. Adolescents who live in areas objectively characterized by high levels of violent crime measured by criminal statistics were no more likely to be distressed than their peers in safer areas.

Aisenberg et al. [43] also did not find an association between community violence and PTSD, and they suggested that other factors, such as one's relationship to the victim and one's physical proximity to the violent event, may influence this association. It is important to underscore that this is the only study included in this review considered low quality. Donenberg et al. [50] did not find an association between community violence and internalizing problems; specifically for externalizing problems in boys, some factors that could have influenced these results are a small sample and the fact that the measurement of community violence considered only witnesses.

The subgroup of 20 studies that were meta-analysed had a summary measure of 1.02 (95% CI 1.01-1.02), showing that there is a small but statistically significant higher risk of internalizing mental health symptoms for adolescents exposed to CV.

### Differences in mental health according to the proximity of CV – victims of CV vs. witnesses of CV

Fourteen studies considered proximity to community violence in the statistical analysis [32, 40, 44, 51, 52, 54, 57, 59, 60, 63, 66–68, 70]. Three of these studies found a gradient risk for mental health outcomes regarding proximity to community violence, which means a larger risk for victims compared to witnessing and/or witnessing compared to merely knowing of violent events [60–68]. Six works found an association for victims of community violence but not for witnesses of community violence, and one found a positive association for different forms of victimization using witnessing as a control [23, 32, 44, 52, 57, 59, 70]. One study found an association between all community violence measures and mental health outcomes with the same magnitude, and three did not find an association either for the victim or for witnessing [32–44, 70, 71, 73].

The results indicate that higher proximity to violence was related to a higher risk for internalizing mental health symptoms. Grinshteyn et al. [54], in addition to a gradient of risk from victims to witnesses to those who merely knew about events, also found differences between violent events and non-violent events, the first one counting for a higher magnitude. The authors that did not find an association discuss the possibility of desensitization and other types of violence (school or family violence) as softening the effects of community violence on mental health [59].

The meta-analysis graphs with victim and witnessing subgroups were not considered because they presented high heterogeneity (61.1% and 67.6%, respectively).

### Assessment of community violence by crime statistics

Six studies measured exposure to community violence with crime rates [31, 35, 48, 53, 54, 71]. Grinshteyn et al. [54] defined crime rates using the crime rate per 1000 people in a given postal code. They also collected self-report data for comparison. Their results pointed to a decreasing gradient risk from victims to witnesses to those who merely knew of violent events. When comparing criminal statistics with self-report measures, the results were positively significant only for depression and at a smaller magnitude. The authors discussed the importance of these area-level crime rates to be constructed in smaller geographic units and to be considered a larger variety of crimes. Goldman-Mellor et al. [53] measured perceived neighbourhood safety with self-respondent answers and objectively measured neighbourhood violence using a geospatial index based on FBI Uniform Crime Reports. Their results showed an association for the first measure but not for the second one, suggesting that perception of neighbourhood violence matters more for mental health than objective levels. Velez-Gomez et al. [71] and Cuartas et al. [48] utilized both criminal statistical analyses and homicide rates. The first group encountered a positive association only for the outcome "ineffectiveness" in early adolescents (10-12 years), and the second group encountered a positive relationship for common mental disorders and post-traumatic stress disorder.

Gepty et al. [35] utilized criminal statistics classifying violent crimes and non-violent crimes and found a positive association with depressive symptoms for the first violent crime but not for non-violent crimes. Da Viera et al. [31], worked with criminal statistics related to adolescents’ residence and school address and found that adolescents who live in areas with low crime and studies in areas with high crime have a larger chance of presenting anxiety, probably related to feelings of insecurity on the way to school.

### Influence of gender, race, and age on the association between CV and internalizing mental health symptoms

Thirteen studies analysed gender as a moderator in the relation above, four of them found gender to be a potential moderator. Bacchini et al. [44] and Boney-McCoy et al. [69] found that girls are more affected by negative experiences of community violence than boys, reacting with high anxiety, depression, sadness and post-traumatic stress symptoms. Haj-Yahia et al. [55] found that girls had more internalizing problems than boys when they were victims of community violence but not witnessing, while Foster et al. [52] found a positive association between community violence and depressive and anxious symptoms only for witnessing but not for victims. The other seven works tested gender as a moderator and did not find differences between boys and girls in the association [35–63].

Only two studies, one conducted in Israel [60] with Arabic and Jewish subjects and another in Chicago [47] with Latinx, Black and White individuals, tested race as a moderator of the relationship between community violence and mental health symptoms. In the first study, Jewish subjects reported higher levels of witnessing community violence, while Arabs reported higher levels of victims of community violence and post-traumatic symptoms over the last year, but this ethnic affiliation did not moderate the relationship between community violence exposure and PTSD. Chen et al. [47] worked with a large multi-ethnic sample in Chicago and found that Latinx and Black adolescents were more exposed to community violence, had higher levels of depression and delinquency, and had more risk factors, such as low family warmth, peer deviance, school adversity and community violence exposure. In addition, the results from regression models showed a higher chance of depression for White adolescents than for minority adolescents (Black and Latinx), which is explained in light of the desensitization hypothesis [77, 78].

The only study that considered age as a moderator of the relationship above was the one conducted by Gomez et al. [71]. Even so, the stratification occurred with an age group that did not fit our inclusion criteria (8-10 years), so the results were presented only for the interval 10-12 years.

### Family support, communication skills, emotional regulation and contextual factors that affect adolescents' mental health when exposed to community violence

Other factors appear to be moderators of the association between community violence and mental health symptoms [26, 44, 56, 58, 63, 65]. Sun et al. [42], O’Leary [64] and Gepty et al. [35]. The most frequent were family characteristics such as mother and father support, parental monitoring, sibling support, and communication skills. Bacchini et al. [44], Howard et al. [58] and Ozer et al. [65] described that parental monitoring/support could reduce depression and symptoms of distress. Talking with their parents and expressing their fears could make young people feel protected, reducing feelings of isolation and danger. Ozer et al. [65] also found that sibling support was protective against post-traumatic stress disorder symptoms and depressive symptoms in adolescents exposed to community violence; teacher help did not have a protective effect on either outcome, and a tendency to keep their feelings to themselves was demonstrated to be a protective factor against post-traumatic stress disorder symptoms [65]. Haj-Yahia et al. [55] and O'Donnell et al. [63] did not find differences in chances of depression and post-traumatic stress disorder for adolescents’ exposure to community violence when family support was present or teacher support for the first.

Individual characteristics of personality and emotional functioning also appear in some studies as moderators. Le Blanc et al. [26] found that good communication and problem-solving skills protect adolescents’ exposure to community violence from psychological stress. Sun et al. [42] encountered that internal dysfunction involving emotional dysregulation, such as self-harm, potentializes symptoms of post-traumatic stress disorder in adolescents exposed to community violence. O’Leary [64] found that expressive suppression, which refers to active inhibition of observable verbal and nonverbal emotional expressive behaviour, buffers the effect of community violence exposure on depression. Gepty et al. [35] studied the ruminative cognitive style, which is the tendency of an individual to be caught in a cycle of repetitive thoughts, and found that it also increases the chance of depression in adolescents exposed to violent crimes.

Contextual factors were also evaluated as moderators. Cuartas et al. [48] studied the effect of living in a poor household, having been directly victimized or witnessing a crime, perceived neighbourhood as unsafe and social support and found that the first three of them potentiate the chance for post-traumatic stress disorder in adolescents’ exposure to community violence and that perceived neighbourhood as unsafe also worsens the chances of common mental disorders. O'Donnell et al. [63] analysed adolescents from The Republic of Gambia, Africa, and found that positive school climate function as a protective factor between community violence exposure and post-traumatic stress disorder, and it was stronger for witnesses than for victims.

Cultural factors related to ethnicity were also evaluated. Henry et al. [56] studied cultural pride reinforcement and cultural appreciation of legacy as potential moderators between community violence and depressive symptoms in a sample exclusively composed of African Americans. Cultural appreciation of legacy was found to be a protective moderator of this relationship, leading to the conclusion that teaching African American youth about their cultural heritage can help them cope with racial discrimination.

### Different risks for different outcomes

Some studies analysed more than one outcome with the following distribution: depression (20), internalizing symptoms (16), post-traumatic stress disorder (15) and anxiety/stress (1). Different outcomes are associated with different magnitudes of community violence exposure, as shown in Table 1, and factors analysed as moderators of this association also act differently.

The graphs of meta-analysis in subgroups by outcome that showed a heterogeneity below 50% and were therefore presented in this review were the studies with post-traumatic stress disorder as outcome and internalizing symptoms. The summary measures for post-traumatic stress disorder outcome were greater than 1 (1.12, 95% CI 1.05–1.19), while for the internalizing symptoms, the outcome was borderline (1.02, 95% CI 1.00–1.04).

## Discussion

The results of qualitative synthesis reinforced a positive relation encountered in the previous meta-analysis between community violence exposure and internalizing mental health symptoms in adolescents [18, 19]. The summary measure from 20 studies in quantitative synthesis showed a small but positive association. The proximity of community violence appeared to be an essential factor contributing to the risk of mental health symptoms. Adolescents who are victims of community violence are at greater risk than those who are witnessing community violence. The summary measures of the victim and witnessing subgroups could not be considered due to the high heterogeneity. Regarding the outcomes analysed, studies showed different risk magnitudes for different outcomes. The summary measures for post-traumatic stress disorder were positive and small but larger than those for the subgroup of internalizing symptoms.

Longitudinal studies provide stronger evidence than cross-sectional studies since they can establish cause and effect relationships [79]. Of the twelve studies with a longitudinal design included in this review, 10 showed at least one significant effect measure in the causal association between greater exposure to community violence and increased risk of developing internalizing mental disorders. This fact supports the idea that there is a causal association in this relationship. Regarding moderators mentioned in objectives (gender, age, and race), only female gender appeared to be a significant moderator in 4 studies. These differences between genders are also found in studies that consider externalizing symptoms; however, for this outcome, boys have more risk than girls when exposed to community violence. A possible explanation for this distinction is the difference in upbringing between boys and girls, especially in more traditional societies, where girls are encouraged to keep their emotions to themselves and to have more socially acceptable behaviour, while the boys are encouraged to reinforce their masculinity, sometimes through violent and deviant behaviour [80].

Age was also not tested in the majority of studies as a possible moderator. In the previous meta-analyses conducted by Fowler [19], which included children and adolescents, differences were found between these two stages of the life cycle, with teenagers having the greatest risk. In regard to teenagers, on the one hand, a tendency towards a greater circulation around the neighbourhood by older adolescents is expected when compared to younger adolescents, which can mean a higher exposure to community violence in the first group. On the other hand, the emotional maturation expected over the years can protect against the effects of violence on mental health. Given the scarcity of studies that assess this influence, we can point out this gap as an area to be researched in future studies. Race was tested as a moderator only in two studies – one with Jews and Arabs and the other with White, Black and Latinx subjects. The former study found that Latinx and Black adolescents are at higher chance of developing depression when exposed to community violence. It is important to highlight the fact that thirteen studies of the forty-two studies included in this review did not have any information about the race of participants. On the other hand, in the group of studies that classified race participants, some of them were composed exclusively of African Americans. It must be pointed out that the lack of this information, as well as the homogeneity of the samples, is an important failure of the studies. Previous meta-analyses could not evaluate race as modifiers because of these same problems [19]. As a counterpoint, a systematic review and meta-analysis showed that racism is linked to poor physical and mental health [81]. Since there is substantial gender inequality among victims of community violence, with boys more like to be victims, and racism is a critical factor that can influence mental health, it is important to study the effects of race on the association of community violence and mental health symptoms, as well as possible protective factors and interventions for this population [14]. The study by Henry et al. [56] is an example of how maternal messages of positive reinforcement of Black culture can protect against depressive symptoms in adolescents of this ethnic group who are exposed to community violence.

An important aspect to be highlighted that appears in our results and in previous meta-analyses is the phenomenon of desensitization. This phenomenon can occur in areas of high levels of community violence. With chronic and recurrent exposure, individuals do not present as many depressive and anxious symptoms after a certain degree of community violence in a process of naturalization of barbarism [73, 77, 78]. This phenomenon should not be interpreted as beneficial, as this naturalization of violence may have negative effects on other outcomes. In relation to externalizing symptoms, for example, aggressive behaviour and delinquency, we can see the opposite effect: there is an increase in these behaviours in a progressive and linear way with an increase in violence.

Most studies included in this review were conducted in the United States of America (27), followed by South Africa (4), Israel (3), Colombia (2), the Republic of Gambia (1), China (1), England (1), Switzerland (1), Italy (1), and Mexico (1). Globally, community violence varies according to region and country. According to the World Health Organization [82], homicide rates were highest in Latin America (84.4/100,000 in Colombia, 50.2/100,000 in El Salvador, 30.2/100,000 in Brazil) and lowest in Eastern European countries (0.6/100,000 in France and 0.9/100,000 in England) and Asia (0.4/100,000 in Japan). In this review, exposure rates to community violence were different between studies. For example, four studies conducted in Africa reported that 83.4% to 98.9% of subjects were witnesses of community violence, while 40.1% to 83.5% of subjects were victims of community violence [59, 63, 66, 70]; in contrast, studies in the United States of America showed greater variation, witness of community violence (49-98%) and victim of community violence (10.3-69%) [26, 32–34, 36–39, 41, 43, 51–54, 56, 58, 62, 65, 68]. Part of this difference could be due to different methods for measuring community violence, but another part could be because of different population origins. Socioeconomic level, social inequalities, urban disorder, weather factors, and cultural factors can influence community violence exposure rates and can also influence how adolescents react to them [83–85]. Therefore, different territories can count on different levels of community violence and different ways to deal with it. Some studies of this review reinforced this aspect; for example, Cuartas et al. [48] studied the effect of contextual factors such as poverty in the neighbourhood and social support as potential moderators of the association of community violence and CMD and PTSD, confirming their hypotheses for the former. O'Donnell et al. [63] found that a positive school climate was a protective factor for youth who witnessed CVs about post-traumatic stress reactions. The authors highlighted the high levels of self-report hostile school climate that may reflect the school context's structural factors. However, considering the cultural aspects, any of the studies included in this review compared, for example, urban areas with rural areas. It would be an interesting comparison to examine. Considering these variations attributed to contextual and cultural factors, more studies conducted in different countries and cities would be relevant.

In this review, some studies analysed the difference between exposure to violence measured by statistical criminalities and community violence self-report questionnaires or perceived violence [53, 54]. The authors found differences in their results, as described in section 3.3. The first methodology has relevance because it is less costly and simpler to conduct and therefore has importance, especially in countries where there are few studies in this area. Nevertheless, studies that compare two forms of measuring violence (self-report and criminal statistics) can contribute to a better understanding of the differences between them.

There are strengths and limitations that should be considered in this systematic review. Strengths include an extensive search of databases, contact with authors for clarification and no filters applied for year or language in the search, all of them contributing to a larger body of literature. Methodologies were constructed with alternate pairs of studies in the selection and extraction phase to avoid selection bias and errors in extraction. Studies included in the review were composed mostly of adolescents from schools or population-based samples and not from mental health services or other types of institutions, leading to a more representative sample. This review utilized a community violence concept that excludes sexual and school interpersonal violence, focusing on and estimating the effect of such violence on adolescents’ mental health, which we considered a strength since it brings more specificity to the results. The main limitations were that different tools for exposure and outcome measures were used, leading to heterogeneous results and compromised pooling. Study designs and statistical analysis also differed between studies, which made comparison difficult.

## Conclusion

This review confirmed a positive relationship between community violence, excluding sexual assault and school violence, and internalizing mental health symptoms in adolescents. Even though race and age did not appear to be moderators in most of the studies, girls were more sensitive to the effects of the exposure in some studies, showing that gender can be a possible moderator in this relationship. Other factors, such as family constitution, communication skills and emotional functioning, also seem to have an influence on this association.

This review provides relevant information regarding the health and public safety field and can serve to direct public efforts to build policies to address the prevention and treatment of both community violence and mental disorders. This review also contributes to knowledge of these issues among health and education professionals.

## Supplementary Information

**Additional file 1.**

**Additional file 2.**

**Additional file 3.**

**Additional file 4.**

## Data Availability

All data generated or analysed during this study are included in this published article (and its supplementary information files).

## List of abbreviations

CI: confidence interval
CV: community violence
LILACS: Literatura Latino-americana e do Caribe em Ciências da Saúde
NI: no information
OR: odds ratio
PRISMA: Preferred Reporting Items for Systematic Reviews and Meta-Analyses
PROSPERO: Prospective Register of Systematic Reviews
PTSD: post-traumatic stress disorder
SD: standard deviation
SE: standard error
USA: United States of America
VCV: victim of community violence
WCV: witness of community violence
