# Supplementary material for: Community violence and internalizing mental health symptoms in adolescents: A systematic review
Source: BMC Psychiatry. 2022 Apr 9;22:253. doi: 10.1186/s12888-022-03873-8 (PMC8994919; doi:10.1186/s12888-022-03873-8)
Supplement: Supplementary file 3 — Additional file 3. [file 12888_2022_3873_MOESM3_ESM.docx]

# Forest plots graphics

# Open " 11_01_2022_database metanalise.xlxs"

# forest plot graphic - total Community violence with 19 studies. Manually removed: Aisenberg (low quality) and Sui + Klodinick (large confidence intervals)

# this forest plot corresponds to FIGURE 2.

**metan orcvgeral1 seorcvgeral1, lcols ( primeiroautor ano)**

# forest plot graphic for post traumatic stress disorder (PTSD)- corresponds to FIGURE 3.

# Open excel file "dados_Claudia.xlsx", sheet("TEPT_medias CVgeral") firstrow case(lower) clear

**metan orcvgeral1 secvgeral1, lcols( author year)texts(150)**

# forest plot graphic for depression - not show in the figures.

# Open excel file "dados_Claudia.xlsx", sheet("depressao_medias CV geral") firstrow case(lower) clear

**metan orcvgeral1 secvgeral1, lcols ( author year)texts(150)**

# forest plot graphic to internalizing symptoms - not show in the figures.

# Open excel file "dados_Claudia.xlsx", sheet("s.intern_médias CV geral") firstrow case(lower) clear

**metan or se, lcols( author year)texts(200)**

# forest plot graphic to internalizing symptoms without study from "Xingcheng sui" (large confidence intervals) - corresponds to FIGURE 4

**metan or se, lcols( author year)texts(200)**
