# Supplementary material for: Community violence and internalizing mental health symptoms in adolescents: A systematic review
Source: BMC Psychiatry. 2022 Apr 9;22:253. doi: 10.1186/s12888-022-03873-8 (PMC8994919; doi:10.1186/s12888-022-03873-8)
Supplement: Supplementary file 4 — Additional file 4. [file 12888_2022_3873_MOESM4_ESM.docx]

APPENDIX I – Search strategy

**Search terms:**

(Adolescents OR Adolescence OR Teens OR Teen OR Teenagers OR Teenager OR Youth OR Youths OR “Female Adolescent” OR “Female Adolescents” OR “Adolescents, Male” OR “Adolescent, Male” OR “Male Adolescent” OR “Male Adolescents")

AND

(“community violence” OR “urban violence” OR “neighborhood violence” OR “victim of violence”)

AND

("mental health" OR "health, mental" OR "mental hygiene" OR "anxiety disorder" OR "disorder, anxiety" OR "disorders, anxiety" OR "anxiety neuroses" OR "neurotic anxiety state" OR "states, neurotic anxiety" OR "common mental disorders" OR aggression OR aggressions OR "juvenile delinquency" OR "substance-related disorders" OR "drug abuse" OR "drug dependence" OR "dependence, drug" OR "drug addiction" OR "addiction, drug" OR "substance use disorders" OR "disorder, substance use" OR "substance use disorder" OR "drug use disorders" OR "drug users" OR "drug use disorder" OR "substance abuse" OR "abuse, substance" OR "substance abuses" OR "substance dependence" OR "substance addiction" OR "addiction, substance" OR "prescription drug abuse" OR "drug abuse" OR "drug habituation" OR "alcohol-related disorders" OR "alcohol related disorders" OR "disorder, alcohol-related" OR "binge drinking" OR "drinking, binge" OR "alcoholic intoxication" OR "intoxication, alcoholic" OR drunkenness OR drunkennesses OR "academic failure" OR "academic failures" OR "failures, academic" OR ptsd OR "stress disorder, post traumatic" OR "posttraumatic stress disorders" OR "posttraumatic stress disorder" OR "stress disorder, posttraumatic" OR "post-traumatic neuroses" OR "post-traumatic stress disorders" OR "post traumatic stress disorders" OR "post-traumatic stress disorder" OR "stress disorder, post-traumatic mental disorder" OR "disorder, mental" OR "disorders, mental" OR "mental disorder" OR "diagnosis, psychiatric" OR "psychiatric diagnosis" OR "behavior disorders" OR "disorders, behavior" OR "internalizing symptoms" OR "externalizing symptoms" OR "conduct disorder" OR "conduct disorders" OR "stress, psychological" OR "psychological stress" OR "psychological stresses" OR "stresses, psychological" OR "life stress" OR "life stresses" OR "stress, life" OR "stresses, life" OR "stress, psychologic" OR "psychologic stress" OR "stressor, psychological" OR "psychological stressor" OR "psychological stressors" OR "stressors, psychological" OR "emotional stress" OR "stress, emotional" OR "mental suffering" OR "suffering, mental" OR suffering OR sufferings OR anguish OR "behavioral symptoms" OR "behavioral symptom" OR "symptom, behavioral" OR "symptoms, behavioral" OR depression OR depressions OR "depressive symptoms" OR "depressive symptom" OR "symptom, depressive" OR "symptoms, depressive" OR "emotional depression" OR "depression, emotional" OR "depressions, emotional" OR "emotional depressions")

Note: The search included terms related to externalizing problems and learning disabilities because at this time of the review, the target outcomes still included mental health conditions other than internalizing symptoms. In the selection phase, it was decided to restrict the outcome to internalizing symptoms.

APPENDIX II – Search conducted on March 9^th^, 2019

| **Database** | **Filters applied** | **Number of results** |
| --- | --- | --- |
| Medline | **- Species**: humans.  **- Article type:** classical articles, clinical studies, comparative studies, journal articles, observational studies.  - **Age** children (6-12 years), adolescents (13-18 years), young adults (19-24). | 188 |
| Web of Science | **- Document type:** articles included, books and reviews excluded. | 987 |
| Embase | **- Age:** school-age children (7-12 years), adolescents, young adults.  - **Study type**: all types except clinical trials, randomized clinical trials, controlled clinical trials, case reports, clinical protocols. | 129 |
| PsycInfo | **- Age**: school-age children (6-12 years), adolescents (13-17 years), young adults (18-29 years).  **- Population**: humans.  **- Methodology**: empirical studies, follow-up studies, longitudinal studies, prospective studies, retrospective studies, quantitative studies.  **- Document type**: dissertations, journal articles. | 727 |
| LILACS | **- Document type**: articles included, congress and conference presentations excluded.  **- Limit:** humans.  **- Study type:** cohort studies, case–control studies, and economic evaluations of health included; case reports, reviews and systematic reviews excluded. | 220 |
| Scopus | **- Document type:** published articles and articles in press included; reviews, book chapters, editorials, and conference presentations excluded. | 388 |

APPENDIX III – Search conducted on January 14^th^, 2021

| **Databases** | **Filters applied** | **Number of results** | |
| --- | --- | --- | --- |
| Medline | - Species: humans.  - Article type: classical articles, clinical studies, comparative studies, journal articles, observational studies.  - Age: children (6–12 years), adolescents (13-18 years), young adults (19–24 years).  - Published in the last 2 years. | 41 | |
| Web of Science | - Document type: articles.  - Publication year: 2019-2021.  - Categories excluded: law or automation control systems, developmental biology, substance abuse, hospitality/leisure, sport, tourism, medical research experiments, neurosciences, pharmacology/pharmacy. | 142 | |
| Embase | - Age: school-age children (7-12 years), adolescents, and young adults.  - Study type: articles, data papers.  - Publication year: 2019-2021 | 49 | |
| PsycInfo | - Age: school-age children (6-12 years), adolescents (13-17 years), young adults (18-29 years).  - Population: humans.  - Methodology: empirical studies, follow-up studies, longitudinal studies, prospective studies, retrospective studies, quantitative studies.  - Document type: dissertations and journal articles. | | 85 |
| LILACS | - Document type: articles.  - Limits: humans.  - Study design: cohort studies, case–control studies, and economic evaluations of health.  - Publication year: 2019-2021. | 2 | |
| Scopus | - Filters: published articles, articles in press, undefined.  - Publication year: 2019-2021. | 82 | |

APPENDIX IV – Critical appraisal tools

**1. Critical appraisal tool for cohort studies:^1^**

*Adapted from Meta-Analysis of Statistical Assessment and Review Instrument (MAStARI)* critical appraisal tools for comparable cohort/case control studies as presented in the *Joanna Briggs Institute Reviewers’ Manual, 2014 Edition.*

- 1. Is the sample representative of patients in the population as a whole?
  2. Are the patients at a similar point in the course of the condition/illness?
  3. Has bias been minimized in selection?
  4. Are confounding factors identified and strategies to deal with them stated?
  5. Was follow-up carried out over a sufficient time period?
  6. Were the outcomes of people who withdrew described and included in the analysis?
  7. Were outcomes measured in a reliable way?
  8. Was appropriate statistical analysis used?
  9. Was the exposure measured in a reliable manner?

**2. Critical appraisal tools for cross-sectional studies:**

*Adapted from (MAStARI)* critical appraisal tools for descriptive/case series studies as presented in the *Joanna Briggs Institute Reviewers’ Manual, 2014 Edition.*

- 1. Was the study based on a random or pseudo-random sample?
  2. Were the criteria for inclusion in the sample clearly defined?
  3. Were confounding factors identified and strategies to deal with them stated?
  4. If comparisons are being made, was there sufficient descriptions of the groups?
  5. Was follow-up carried out over a sufficient time period?
  6. Were outcomes measured in a reliable way?
  7. Was appropriate statistical analysis used?
  8. Was the exposure measured in a reliable manner?

**Adaptations:**

- The question “Was the exposure measured in a reliable manner?” was added to both instruments.

- The question “Has bias been minimized concerning the selection of cases and controls?” was replaced with “Has bias been minimized in selection?” in the second instrument.

- The question “Are outcomes assessed using objective criteria?” was removed. As our analysis did not include any case–control studies, it was assumed that the question “Are outcomes measured reliably?” was sufficient for cross-sectional and cohort studies.

- The question “Was follow-up carried out over a sufficient time period?” was removed from the second instrument because this review did not include any case series.

**Possible answers and scores:** Y – Yes (3 points)

N – No (1 point)

U – Undefined (2 points).

**Classification of quality:**

Low – score 9-15 (cohort studies)/8-13 (cross-sectional studies)

Intermediate – score 16-21 (cohort studies)/score 14-18 (cross-sectional studies)

High – score 22-27 (cohort studies)/score 19-24 (cross-sectional studies)
